# A systematic review and metanalysis of questionnaires used for auditory processing screening and evaluation

**DOI:** 10.3389/fneur.2023.1243170

**Published:** 2023-08-08

**Authors:** Myrto Samara, Hung Thai-Van, Martin Ptok, Eleni Glarou, Evelyne Veuillet, Simone Miller, Pierre Reynard, Helen Grech, Nattawan Utoomprurkporn, Afroditi Sereti, Doris-Eva Bamiou, Vasiliki Maria Iliadou

**Affiliations:** ^1^Department of Psychiatry, Faculty of Medicine, University of Thessaly, Larisa, Greece; ^2^Department of Psychiatry and Psychotherapy, School of Medicine, Technical University of Munich, Munich, Germany; ^3^Institut de l'audition, Institut Pasteur, Inserm, Paris, France; ^4^Université Claude Bernard Lyon 1, Villeurbanne, France; ^5^Service d’Audiologie et d’Explorations Otoneurologiques, Hôpital Edouard Herriot, Hospices Civils de Lyon, Lyon, France; ^6^Department of Phoniatrics and Pedaudiology, Hannover Medical School, Hannover, Germany; ^7^Centre for Trials Research, Cardiff University, Cardiff, United Kingdom; ^8^Division of Population Medicine, School of Medicine, Cardiff University, Cardiff, United Kingdom; ^9^Faculty of Health Sciences, University of Malta, Msida, Malta; ^10^Faculty of Medicine, Chulalongkorn University, Bangkok, Thailand; ^11^Ear Institute, University College London, London, United Kingdom; ^12^Chula Neuroscience Center, King Chulalongkorn Memorial Hospital, Bangkok, Thailand; ^13^Clinical Psychoacoustics Laboratory, 3rd Psychiatric Department, Neurosciences Sector, Medical School, Aristotle University of Thessaloniki, Thessaloniki, Greece; ^14^National Institute for Health and Care Research, University College London Hospitals, UK Biomedical Research Centre, Deafness and Hearing Problems Theme, London, United Kingdom; ^15^Department of Neuro-otology, University College London Hospitals, London, United Kingdom

**Keywords:** auditory processing disorders, auditory processing, central auditory processing, hearing, questionnaires, clinical test battery, systematic review, metanalysis

## Abstract

The recognition of Auditory Processing Disorder (APD) as a distinct clinical condition that impacts hearing capacity and mental health has gained attention. Although pure tone audiometry is the gold standard for assessing hearing, it inadequately reflects everyday hearing abilities, especially in challenging acoustic environments. Deficits in speech perception in noise, a key aspect of APD, have been linked to an increased risk of dementia. The World Health Organization emphasizes the need for evaluating central auditory function in cases of mild hearing loss and normal audiometry results. Specific questionnaires play a crucial role in documenting and quantifying the difficulties faced by individuals with APD. Validated questionnaires such as the Children’s Auditory Processing Performance Scale, the Fisher’s Auditory Problems Checklist, and the Auditory Processing Domains Questionnaire are available for children, while questionnaires for adults include items related to auditory functions associated with APD. This systematic review and meta-analysis identified six questionnaires used for screening and evaluating APD with a total of 783 participants across 12 studies. The questionnaires exhibited differences in domains evaluated, scoring methods, and evaluation of listening in quiet and noise. Meta-analysis results demonstrated that individuals with APD consistently exhibited worse scores compared to healthy controls across all questionnaires. Additionally, comparisons with clinical control groups showed varying results. The study highlights (i) the importance of standardized questionnaires in identifying and assessing APD, aiding in its diagnosis and management, and (ii) the need to use sub-scores as well as overall scores of questionnaires to elaborate on specific hearing and listening situations. There is a need to develop more APD specific questionnaires for the adult population as well as for more focused research on APD diagnosed individuals to further establish the validity and reliability of these questionnaires.

## 1. Introduction

The recent report from the World Health Organization (WHO) (1) on hearing includes Auditory Processing Disorder (APD) as a distinct clinical condition that degrades hearing capacity. The report recognizes the existence of APD throughout the lifespan and its negative impact on mental health (1). While pure tone audiometry remains the gold standard for assessing hearing capacity, there is significant scientific evidence indicating that this gold standard inadequately reflects everyday hearing abilities (2), especially in challenging acoustic environments. Additionally, deficits in speech perception in noise, which are a key clinical aspect of APD, have been identified as an independent predictor of an increased risk of dementia (3). Notably, the WHO (1) report emphasizes the need for evaluating central auditory function (i.e., auditory processing) in cases of mild hearing loss, as well as in individuals with normal pure tone audiometry results, in line with the European APD consensus (4). This consensus highlights that APD is a common type of hearing impairment that often goes unrecognized and under-investigated, despite its significant impact on communication, social interactions, emotional well-being, and academic/work performance, ultimately affecting community inclusion.

The main issues in APD are difficulties with hearing in acoustically challenging environments or when faced with complex auditory tasks in real-life situations, even in the absence of significant audiometric findings. It is crucial to document and quantify these difficulties, and this can be achieved using specific questionnaires. These questionnaires are a key component in the evaluation of APD as they guide the implementation of an auditory processing test battery based on the patient’s questionnaire responses, together with their medical and neurodevelopmental history, and their specific needs and inform their management (5).

Examples of validated questionnaires available for children with APD include the Children’s Auditory Processing Performance Scale (CHAPPS) (6), the Fisher’s Auditory Problems Checklist (FAPC) (7), and the Auditory Processing Domains Questionnaire (APDQ) (8). These questionnaires provide information on various aspects of auditory function that are either directly related to hearing (such as performance in quiet, ideal situations, or in noise for CHAPPS) or indirectly related to hearing (such as attention and memory).

For adults, the questionnaires on hearing were not developed to specifically focus on APD, but they include questions directly related to difficulties in a range of auditory functions subserved by the auditory brain and associated with APD. Previous research has demonstrated that these questionnaires provide information on patient symptoms that correlate with APD tests, allowing differentiation between normal controls, individuals with hearing loss, and those with APD (9).

The aim of the current study is to evaluate the ability of existing questionnaires on APD in children and adults to separate APD diagnosed individuals from normal controls as well as from other clinical groups with normal auditory processing.

## 2. Materials and methods

This systematic review and meta-analysis follows PRISMA guidelines (10) and was registered in the PROSPERO database (registration number CRD42021234166) database for the International Prospective Register of Systematic Reviews. The aim of this review was to identify the questionnaires used on individuals diagnosed with APD to assist in screening for the disorder and optimize the test battery for the evaluation of APD. As questionnaires enquiring about hearing difficulties can be used both as screening tools and as auxiliary tools during diagnostic assessments, both uses will be included. Three databases (Scopus, PubMed, Cochrane) were searched using the keywords {[(auditory) AND (processing) AND (disorder)] OR [(central) AND (auditory) AND (processing) AND (disorder)] AND (questionnaire)}. The initial search was conducted on March 13th, 2021 and was last updated on May 31st, 2023. To be included, a study needed to diagnose APD in line with the gold standard approach for diagnosing the disorder. Articles that presented participants and groups with clear diagnostic criteria, such as those from the European APD consensus (11), American Academy of Audiology (AAA) (12), American Speech-Language-Hearing Association (13), and International Bureau of Audiophonology (BIAP) (14), were included. These diagnostic criteria are used in international guidelines to ensure that the individuals and groups described meet specific diagnostic criteria and are not simply suspected of having APD. Diagnostic criteria for APD are based on abnormal results defined as more than two standard deviations from the mean in standardized auditory processing tests for at least one ear including non-verbal tests. Articles that presented data on mixed populations, including both diagnosed and undiagnosed individuals, were excluded from this systematic review as they are not considered specific to APD.

Titles and abstracts, as well as full texts, when necessary, were screened in a pair-wise manner by two independent reviewers, one senior and one younger researcher. There was no language restriction applied during the screening of titles and abstracts. However, for the full-text evaluation, only articles in languages spoken by the authors were considered. In cases where there was a conflict between the two authors regarding a specific article, a third senior author provided input to resolve the conflict and make a decision on whether to include the article.

Subsequently, the resulting papers were critically appraised using the checklist for analytical cross-sectional studies (15), a critical appraisal Joanna Briggs Institute (JBI) that includes eight questions. The critical appraisal aimed to assess the methodology quality (internal validity) and the risk of bias (external validity and generalization of results). The appraisal tool assigns one point for each “yes” answer, resulting in a total score of 8 if all questions are positively answered (15). To provide a more analytical approach and align the critical appraisal with the area of APD, the authors further agreed to document the specific elements of the different questionnaires as reported in the studies. These elements included the domains evaluated, questionnaire scoring, whether an evaluation of comparison between listening in quiet and listening in noise was included, and whether the measurement of reliability and validity of each questionnaire was reported.

### 2.1. Statistical analysis

For studies involving non-APD clinical and/or healthy populations as a control arm, a pairwise meta-analysis was conducted to provide a more comprehensive description and visualization of score differences in the various questionnaires between the groups. The effect size used was the standardized mean difference, expressed as Hedges’ adjusted g. The pooling of studies utilized the standard inverse variance weighting method. Considering the anticipated heterogeneity among the studies, the Der-Simonian and Laird random-effects model was consistently employed (16). Meta-analytic calculations were performed using RevMan 5.4 (17).

## 3. Results

**Figure 1:**
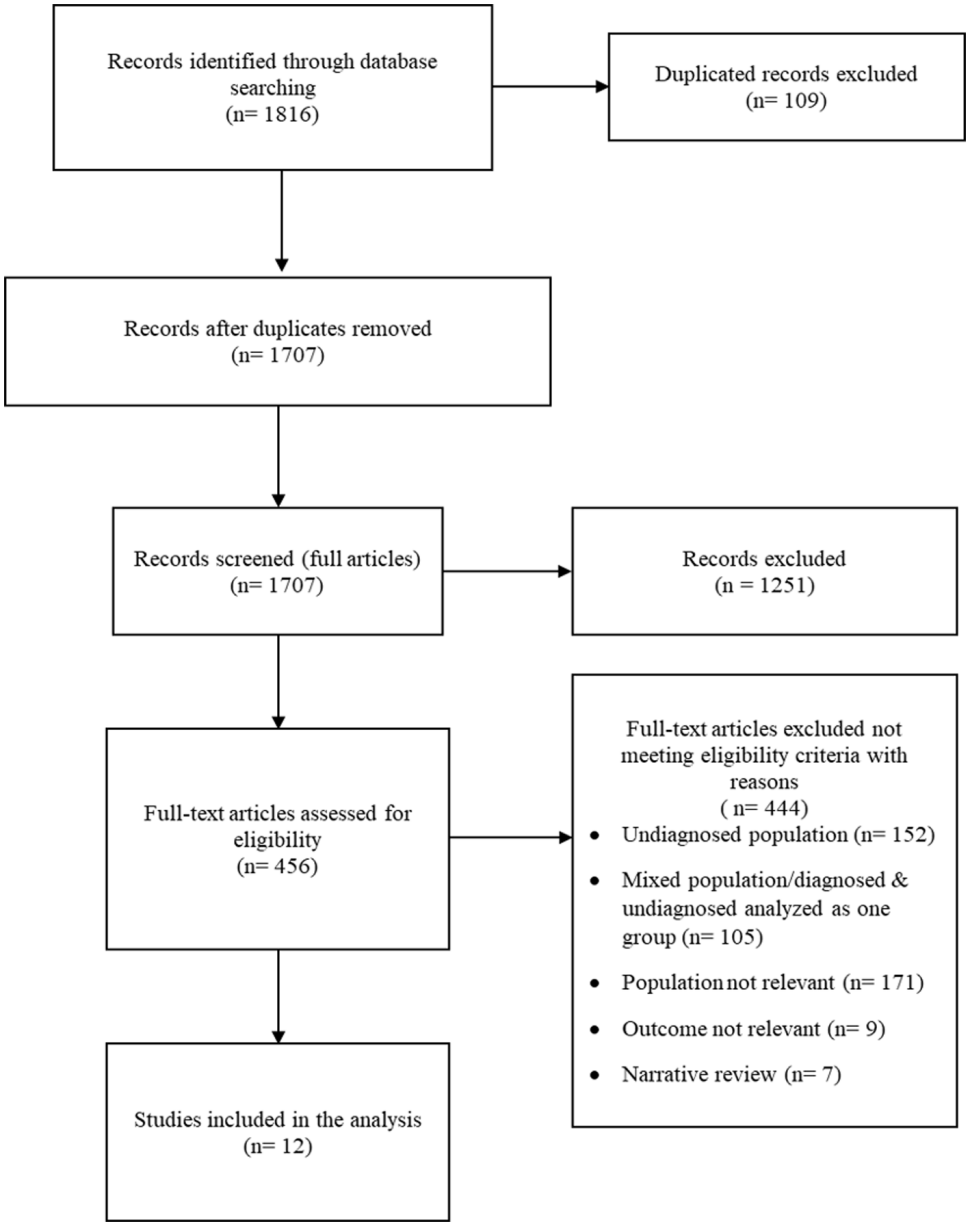
PRISMA flow diagram outlining selection of relevant studies.

The search resulted in the identification of 1816 articles. 109 duplicates were automatically found. For each of the remaining 1707 articles, titles/abstracts were screened and 1,251 were excluded. 456 full text articles were reviewed. At the end, 12 studies with 266 unique participants with APD, 129 participants with other clinical conditions and 388 healthy controls were identified through the literature search. Studies were published from 2008 to 2020. The PRISMA flowchart is shown in Figure 1.

Six questionnaires were found as a result of this systematic review. Five of the questionnaires (i.e., CHAPPS, AIAD, FAPC, SSQ, APDQ) evaluated listening difficulties and other symptoms related to APD and the sixth (i.e., Hyperacusis Questionnaire [HQ]) specifically evaluated the symptom of hyperacusis that may be present in APD. Scores in different studies are presented per examined questionnaire in Tables 1–6. The CHAPPS is a questionnaire primarily designed for parental feedback on hearing difficulties, with the option to be given to teachers to provide feedback on a child’s behavior at school. It consists of six subscales: perception in noise, in quiet, in ideal (one-on-one) situations, multiple inputs, memory, and attention (6). The first four subscales directly assess hearing-related difficulties, while the last two evaluate cognitive skills indirectly related to hearing. The noise and quiet subscales of CHAPPS each have 7 questions, the ideal and multiple inputs subscales have 3 questions each, and the memory and attention subscales contain 8 questions each. Responders are asked to rate the child’s hearing difficulties relative to children of the same age using the following scale: less difficulty (+1), same amount of difficulty (0), slightly more difficulty (−1), more difficulty (−2), considerably more difficulty (−3), significantly more difficulty (−4), and cannot function at all (−5) (6). A raw score is calculated by summing the scores of individual question items, providing an overall result that can be divided by 7 to obtain an average overall score. Raw scores are also available for each subscale, which can be divided by the number of questions in each subscale to derive an overall score for that specific subscale. This questionnaire is utilized for both screening and complementing the diagnostic auditory processing evaluation.

**Table 1:** CHAPPS (pediatric): scores of included studies.

Article	Mean APD parent	sd APD	Mean APD teacher	sd Teacher	n APD	Mean normal control	sd normal control	n Normal control	Mean Clinical control	sd Clinical control	n Clinical group
Dawes and Bishop (18)	−2.09	0.77			25				−1.6	0.67	19 dyslexia
Dawes et al. (19)	−2.1	1			10				−2	2.9	21 non-APD but referred for AP evaluation
Iliadou and Bamiou (20)	−1.8 n −0.8 q −0.7 i −1.2 m −2.2 me −1.1a total = −7.9	0.9 n 0.8 q 1.1 i 0.9 m 1.2 me 0.9 a total = 3.9			38	−0.6n −0.3q 0.1i −0.3 m −0.5me −0.2a total = −1.8	0.4n 0.3 q 0.5 i 0.6 m 0.5 me 0.4 a total = 1.7	39	−2.1 n −0.1 q 0.1 i −1.9 m −0.4 me −0.5 a total = −5.1	0.3 n 0.1 q 0.3 i 1 m 0.3 me 0.4 a total = 1.2	20 non-APD but referred for AP evaluation
Kuk et al. (21)	−2.3 n −1,3 q −0.4 i −0.8 m −2.6 me −1,6 a total = −6.6	0,9 n 0,8 n 0.9 i 0.8 m 1 me 0.9 a total = 0.8	−1.6 n −1.2 q −0.6 i −0.7 m −1.4 me −0.4 a total = −5.9	1.4 n 0.9 q 1 i 0.7 m 1.1 me 1 a total = 1	Children = 17 Parents = 17 Teachers = 17						
Loo et al. (22)			−0.78	0.76	Children = 20 Teachers = 20						
Shaikh et al. (23)	−8.9	not provided			44						

APD, Auditory Processing Disorder; a, attention; i, ideal; me, memory; m, multiple inputs; n, noise; sd, standard deviation; total, total of 6 categories for 36 questions (in total); q, quiet.

**Table 2:** AIAD (adults): scores of included studies.

Article	Mean APD	sd APD	n APD	Mean Normal control	sd Normal control	n Normal control	Mean Clinical control	sd Clinical control	n Clinical group
Bamiou (9)- category 1	56 (2 score x 28 total qus) subscales: SiN 7.5 (1.5×5) SiQ 9.5 (1.9×5) Loc 9.5 (1.9×5) Dist 18.4 (2.3 × 8) Det 11 (2.2 ×5)	0.65 total 0.67 SiN 0.63 SiQ 0.88 Loc 0.75 Dist 0.73 Det	39	75.6 (2.7 score x 28 total qus) subscales: SiN 13 (2.6×5) SiQ 13.5 (2.7×5) Loc 13.5 (2.7×5) Dist 22.4 (2.8 × 8) Det 13.5 (2.7 ×5)	0.3 total 0.56 SiN 0.4 SiQ 0.3 Loc 0.28Dist 0.33 Det	30	67.2 (2.4 score x 28 total qus) subscales: SiN 9 (1.8×5) SiQ 12 (2.4×5) Loc 11 (2.2×5) Dist 21.6 (2.7 × 8) Det 11.5 (2.3 ×5)	0.4	19 non-APD but referred for AP evaluation
Koohi et al. (24) – category 1	66.21	12.98	24	76.22	9.46	18			

APD, Auditory Processing Disorder; qus, questions.

**Table 3:** FAPC (pediatric): scores of included studies.

Article	Mean APD parent	sd APD	Mean APD teacher	sd Teacher	n APD	Mean normal control	sd Normal control	n Normal control	Mean Clinical control	sd Clinical control	n Clinical group
Cameron (25) – category 2	48	16			10	91.6	10.9	50			
Dawes (19) – category 1 retrospective	48	26.4	Not clear if the questionnaire was filled by parents or teachers		8				47.8	16.9	21 non-APD but referred for AP evaluation
Shaik (23) -category 2	Data are presented added together as in median 58 (min 8, max 100, Q1 40, Q3 72)				44 (7-24y)			41 (7-24y) both pediatric and young adult			

AP, auditory processing; APD, Auditory Processing Disorder; Q, quartile; y, years.

**Table 4:** SSQ (pediatric [1] and adults [1]): scores of included studies.

Article	Mean APD	sd APD	n APD	Mean Normal control	sd Normal control	n Normal control	Mean clinical control	sd Clinical control	n Clinical group
Bamiou (9) – category 1	4.7 spch 5.9 spt 6.1 qlt total = 5.6	2.2 spch 2.8 spt 2.1 qlt total = 2.2	39	7.8 spch 8.1 spt 7.9 qlt total = 8	2 spch 2 spt 1.9 qlt total = 1.9	30	5.8 spch 6.8 spt 6.9 qlt total = 6.5	1.4 spch 1.6 spt 1 qlt total = 1.2	19 (failing 1 test of APD established battery)
Cameron and Dillon (26)	1.47 q 3.10 n	0.48 q 0.40 n	9 with spatial processing disorder were given the pediatric SSQ The Speech, Spatial and Qualities of Hearing Scale for Children with Auditory Processing Disorder Unpublished pediatric adaptation of Noble and Gatehouse, 2004 12 questions (no clear cut-off is provided, this was not tested in a normal group)						

APD, Auditory Processing Disorder; n, noise; spt, spatial; spch, speech; sd, standard deviation; q, quiet.

**Table 5:** HQ (adults): scores of included studies.

Article	Mean APD	sd APD	n APD	Mean Normal control	sd Normal control	n Normal control	Mean Clinical control	sd Clinical control	n Clinical group
Bamiou (9) – category 1	21	9.5	39	8.2	5.7	30	17	8.6	19 (failing 1 test of APD established battery)
Spyridakou (27) – category 1	23.5	10.8	10	9.2	6.1	12			

APD, Auditory Processing Disorder; sd, standard deviation.

**Table 6:** APDQ (children – adolescents): scores of included studies.

Article	Mean APD	sd APD	n APD	Mean Normal control	sd Normal control	n Normal control	Mean Clinical control	sd Clinical control	n Clinical group
O’Hara and Mealings (28) – category1	38 (y) 43 (o)	14 (y) 22 (o)	20 (10 y, 10o)	82 (y) 87 (o)	16 (y) 14 (o)	198 (104 y 7–10, 94 o 11–17)	ADHD 52 (y), 50 (o) LD 25 (y), 28 (o)	ADHD 18 (y), 20 (o) LD 2 (y), 13 (o)	ADHD 40 LD 10

ADHD, Attention Deficit Hyperactivity Disorder; APD, Auditory Processing Disorder; LD, learning disability; o, older; sd, standard deviation; y, younger.

The Amsterdam Inventory for Auditory Disability and Handicap (AIAD) consists of 30 questions (29), with a modified version (m)AIAD containing 28 questions in total (30). It assesses perceived hearing difficulties in adults across five subdomains: speech in noise (5 questions), speech in quiet (5 questions), auditory localization (5 questions), sound recognition (8 questions), and sound detection (5 questions) (30). Respondents mark their responses as “almost never” (0 points), “occasionally” (1 point), “frequently” (2 points), or “almost always” (3 points). It is important to note that “almost always” indicates no perceived hearing difficulties, while “almost never” indicates perceived hearing difficulties. The overall score is calculated by summing the points based on the marked responses. Additionally, scores are obtained for each subdomain by summing the marked responses within that specific subdomain. This questionnaire can be used for screening an individual’s hearing capacity, as well as assisting in the diagnosis and rehabilitation of hearing difficulties.

The FAPC is a pediatric checklist consisting of 25 items that assess auditory behaviors (7). Each unchecked item (statement) is assigned a score of 4%. If the total score is below 72%, it is recommended to refer the individual for an audiological evaluation of auditory processing (7). The checklist is designed as a screening tool.

The Speech, Spatial, and Qualities of Hearing Scale (SSQ) is a questionnaire that evaluates perceived auditory disability in complex real-life situations (31). It comprises three distinct sections: speech hearing (14 questions), spatial hearing (17 questions), and sound hearing (19 questions). Each question is scored on a scale of 0 to 10, with 0 indicating complete inability and 10 indicating high ability (31). The SSQ provides a total resulting score, as well as three distinct resulting scores for each section, differentiating speech, spatial, and sound as elements of hearing and listening.

The HQ consists of two parts (32). The first part includes three questions on noise exposure and hearing issues, while the second part contains 14 questions. The questionnaire is structured into three subcategories: attention (4 questions), social (6 questions), and emotional (4 questions). The response scale is a 4-point scale, ranging from 0 points indicating no symptoms to 3 points indicating a lot of symptoms (32).

The APDQ is designed to screen for APD in children aged 7–17 years, and it can be completed by parents or teachers (8). The questionnaire consists of 52 items, which are divided into three scales: Auditory Processing (AP) scale with 30 questions, Attention Control (ATT) scale with 9 questions, and Language scale (Lang) with 10 questions. One question overlaps between the AP and ATT scale, one question overlaps between the AP and Lang scale, and 2 questions are used to compare listening in quiet versus listening in noise (8).When rating by the parent or teacher, a behavior is attributed four points if it is performed regularly (>3/4 of the time), three points if it is performed often (1/2–3/4 of the time), one point if it is performed sometimes (1/4–1/2 of the time, indicating a transition between competency and incompetency), and zero points if it is performed rarely (<1/4 of the time). Scale scores are calculated as (sum of item scores in the scale) / (4 × the number of items in the scale) × 100 (8). Scores above 75% suggest skill competency, while scores below 25% suggest skill incompetency.

A common aspect of all questionnaires is the dual scoring approach of one total result adding difficulties in a combined way as well as of sub-scores for different subdomains/functions of hearing and listening. Most of the publications meeting the criteria for being evaluated in this review present data for the total scoring approach alone.

### 3.1. JBI critical appraisal checklist for analytical cross-sectional studies

Table 7 presents the critical evaluation of included studies and Table 8 of specific elements of the different questionnaires. 8 of the included studies were rated as being at high risk for at least one type of bias, with the most commonly observed weaknesses relating to the validity and reliability of outcome measurement as well as the level of appropriateness of statistical analysis used.

**Table 7:** The JBI critical appraisal checklist for analytical cross-sectional studies (15): included studies.

Article	Were the criteria for inclusion in the sample clearly defined?	Were the study subjects and the setting described in detail?	Was the exposure measured in a valid and reliable way?	Were objective, standard criteria used for measurement of the condition?	Were confounding factors identified?	Were strategies to deal with confounding factors stated?	Were the outcomes measured in a valid and reliable way?	Was appropriate statistical analysis used?	Total score
Bamiou (9)	1	1	1	1	1	1	1	1	8
Cameron and Dillon (26)	1	1	1	0	0	0	1	1	5
Cameron et al. (25)	0	1	0	1	0	0	1	0	3
Dawes and Bishop (18)	1	0	0	0	1	0	0	0	2
Dawes et al. (19)	1	0	0	0	1	1	0	0	3
Iliadou and Bamiou (20)	1	1	1	1	1	1	1	1	8
Koohi et al. (24)	1	1	1	1	1	1	0	1	7
Kuk et al. (21)	1	1	0	1	1	0	1	0	5
Loo et al. (22)	1	1	1	0	1	1	0	1	6
O’Hara and Mealings (28)	1	1	1	1	1	1	1	1	8
Shaikh et al. (23)	1	1	1	1	1	0	1	0	6
Spyridakou et al. (27)	1	1	1	1	1	1	1	1	8

**Table 8:** Specific elements of the different questionnaires included.

Questionnaire name	Domains	Scoring	Comparing listening in quiet *vs* listening in noise	Reliability and validity measured
CHAPPS	6	Both overall and domain specific	yes	yes
AIAD	5	Both overall and domain specific	yes	yes
FAPC	0	Overall	no	no
SSQ	3	Overall and domain specific	yes	yes
HQ	3	Overall	no	yes
APDQ	3	Overall and domain specific	yes	yes

AIAD, Amsterdam Inventory for Auditory Disability and Handicap; APDQ, Auditory Processing Domains Questionnaire; CHAPPS, Children’s Auditory Processing Performance Scale; FAPC, Fischer’s Auditory Problems Checklist; HQ, Hyperacusis Questionnaire; SSQ, Speech, Spatial, and Qualities of Hearing Scale.

### 3.2. APD group versus healthy control group

**Figure 2:**
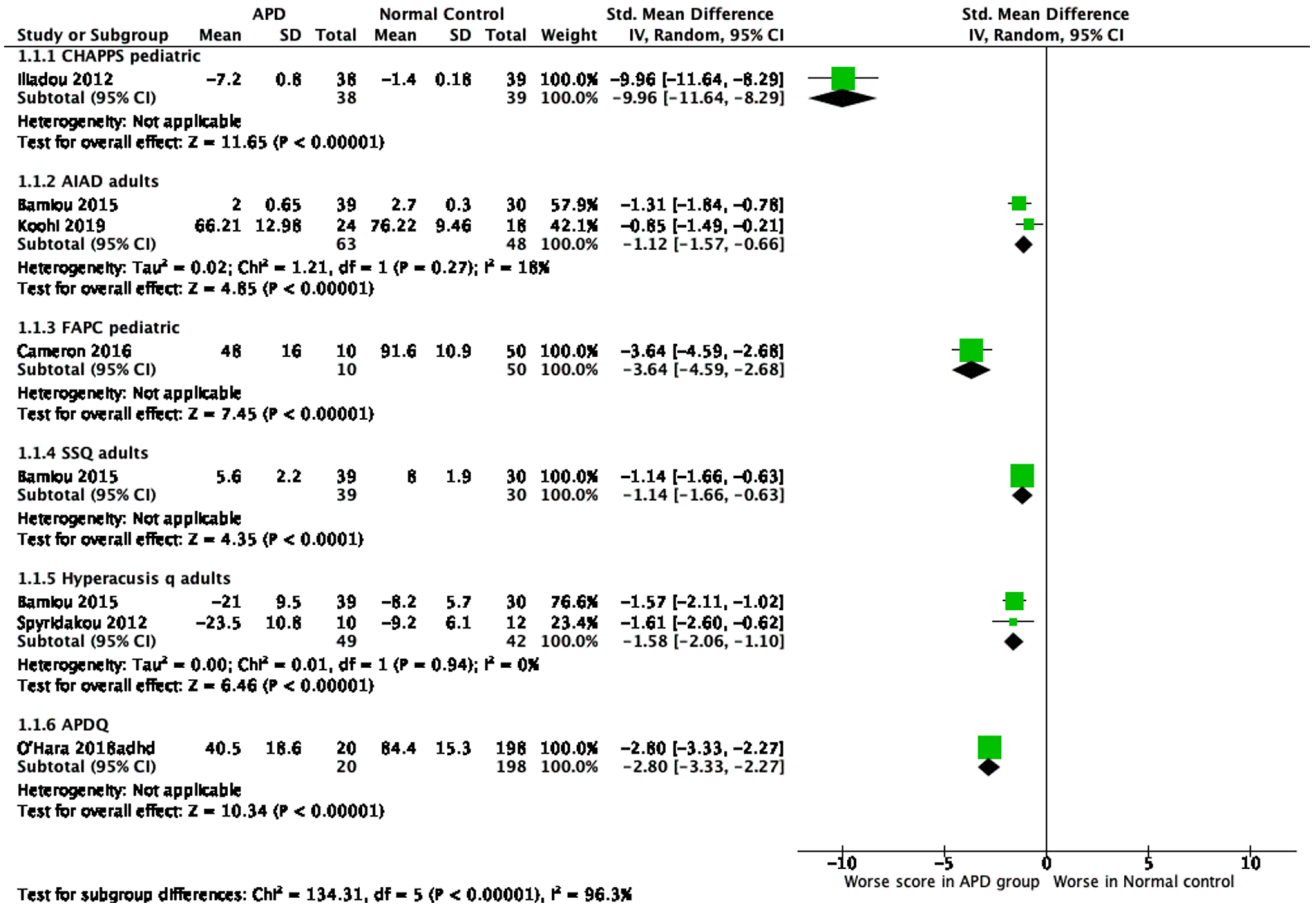
Comparison of symptom severity between APD group versus healthy control group.

Comparisons of symptom severity between APD and healthy control groups, as measured by different questionnaires, are presented in Figure 2. As expected, participants with APD exhibited worse scores compared to the healthy control groups, irrespective of the questionnaire used.

### 3.3. APD group versus clinical control group

**Figure 3:**
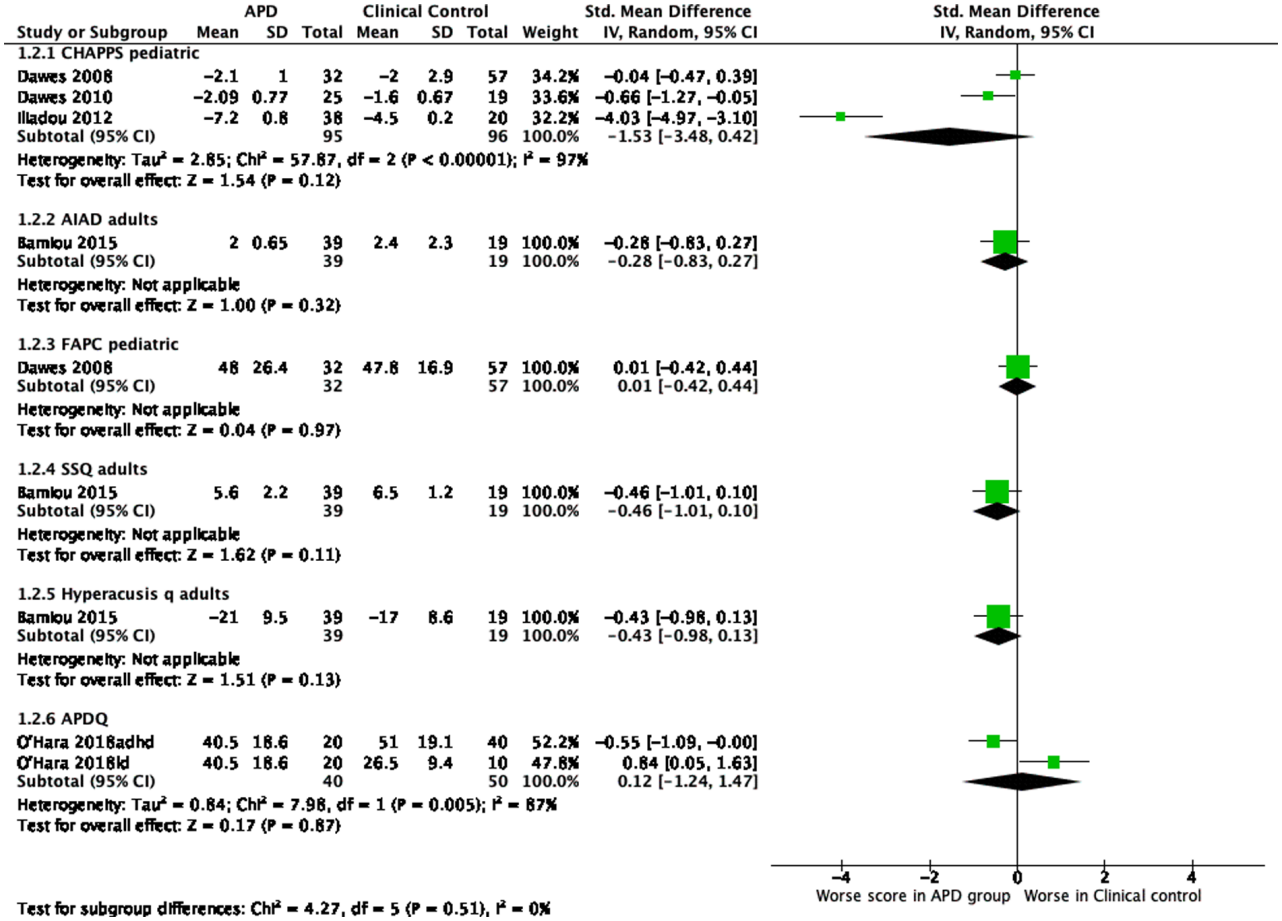
Comparison of symptom severity between APD group versus clinical control group.

Comparisons of symptom severity between APD and clinical control groups are presented in Figure 3. Most clinical control groups included non-APD participants who were referred for AP evaluation but did not meet full criteria for a diagnosis. Scores on the CHAPPS and AIAD questionnaires were worse in participants with APD compared to the clinical control groups. Regarding the APDQ, O’Hara et al. used two different clinical control groups: one with attention deficit hyperactivity disorder (ADHD) and one with learning disabilities. They found that APD participants had worse scores than the ADHD group but better scores than the group with learning disabilities. The remaining questionnaires did not differentiate between the two groups.

## 4. Discussion

This study attempted to capture all the available evidence on the questionnaires applied on individuals diagnosed with APD to provide insights on how efficiently they separate (i) APD diagnosed individuals from normal controls and (ii) APD diagnosed individuals from other clinical non-APD individuals. Six questionnaires, published from 2008 to 2020, were identified with a total of 783 participants across 12 studies. 10 studies (9, 18–25, 28) involved five questionnaires (CHAPPS, AIAD, FAPC, SSQ, APDQ) that evaluate listening difficulties and other APD related symptoms, while the HQ that evaluates the hyperacusis symptom that might occur in APD was involved in two studies (9, 27). The three pediatric questionnaires (CHAPPS, FAPC, APDQ) were designed specifically for APD, whereas the three adult questionnaires were designed for hearing difficulties in general including APD related symptoms and problems. The studies systematically reviewed included more children (226 in total) than adults (160 in total). This indicates a need for specifically designed questionnaires on APD for adults in addition to more research of APD in the adult population. One of the reasons for the limited APD research on adults is the frequent comorbidity of hearing loss that requires more careful clinical assessment to separate issues that might be explained by peripheral hearing loss from those additional issues due to untreated for years hearing loss leading to “auditory deprivation” type plastic changes in the auditory brain (33). The negative impact on cognition that might stem from peripheral hearing loss, an auditory processing disorder or their co-existence is still ignored as revealed by a recent critical review (34). This review reports on P300 being evaluated as a cognition index with no peripheral hearing evaluation in 64% of published studies; of the remaining studies 70% used self-report to rule out hearing loss. It is being concluded that despite strong evidence that hearing loss may interact with and potentiate dementia related pathology (35) as well as present with milder cognitive decline (36, 37) this is often ignored when deciding on the cognitive aspect of a presentation.

It should be noted that most published papers report on the total scores of the questionnaires even in cases where there are sub-scores that could provide more specific information regarding hearing in quiet vs. hearing in noise or auditory attention as well as attention span. This leads to questionnaires possibly being depicted as less efficient than they really are. All questionnaires are depicted as being efficient in separating APD diagnosed individuals from normal controls with a strong effect size (Figure 2), while some of them (CHAPPS, AIAD, SSQ, HQ) seem to be efficient in separating APD diagnosed individuals from other clinical non-APD groups with a small to medium effect size (Figure 3). The clinical non-APD groups are in most cases heterogeneous groups of individuals having similar symptoms to APD, but they are not always provided with specific diagnosis such is the case in the APDQ study. This fact may lead to differences in the presented efficiency of the questionnaires of this systematic review.

Debate on APD (38), while attempting to shed light to the diagnosis and nature of the disorder, has led to more published studies talking about APD, but not diagnosing it. There are several controversies about APD that may involve: contradictory definitions (39), various classification profiles (40), difficulties in differential diagnosis procedure (4), and lack of standardized guidelines (41). Recent diagnostic consensus papers and audiology societies guidelines (11, 12, 14, 42, 43) and inclusion in International Classification of Diseases and Related Health Problems (11th ed.; ICD-11) (44) as AB5Y may help resolve these issues, as indicated by the present review results. The observed questionnaire efficiency for both adult and pediatric questionnaires in separating those with APD from normal or other clinical groups indicates that current guidelines to base diagnosis on psychoacoustic evaluation results according to specific criteria are supported by the level of hearing difficulties reported by the affected individuals, thus providing face validity for the diagnostic approach. This outcome also provides some evidence for the notion that APD when diagnosed by appropriate tests and guidelines is a separate clinical entity to other developmental conditions (i.e., construct validity). It should be noted, however, that a very small number of studies and consequently participants were included in this meta-analysis due to limited publications focusing on APD diagnosed groups. This indicates the need for more research to be conducted on individuals who are formally diagnosed with APD to further confirm or refute the present study results.

As difficulty hearing in noise in the presence of normal hearing in quiet is a core APD symptom, it is essential for a questionnaire on APD to measure this and facilitate the distinction. Four out of the six questionnaires identified have separate sub-scores or elements to provide information on hearing in quiet vs. hearing in noise. However, two of them (i.e., FARP and HQ) do not have items or sub-scores for this distinction. This leads to less granular data that may separate perceived hearing sensitivity related disability vs. perceived auditory processing related disability. The remaining four questionnaires that include these data and can make the distinction are rarely represented in detail in the publications as this systematic review shows.

There are some inherent limitations in this study. Answers regarding difficulties in everyday communication may be over-or underestimated by different individuals based on personality characteristics (45). Auditory based communication difficulties may sometimes be attributed to other factors, i.e., attention, motivation, interest, focus (38). Conversely, some individuals overstress the auditory origin of their communication difficulties (46). Thus, a questionnaire may not well depict the degree or nature of hearing difficulties. A formal diagnosis of APD should typically involve a qualified professional and a multidisciplinary approach, including audiological assessments and behavioral tests, with questionnaires as an additional tool that can provide information about individual experiences and challenges faced with in daily functioning. The information provided may guide choice of tests and additional assessments for the affected individual (47) as well as choice of strategies to address their needs in their everyday life (5).

## 5. Conclusion

A systematic review of a total of 266 APD diagnosed individuals across 12 studies shows that hearing difficulties documented on six different questionnaires are in good agreement with APD diagnosis based on psychoacoustic evaluation and separates affected individuals well from both normal controls and other clinical groups. Limited data exist on reporting questionnaires subscores corresponding to specific hearing and listening situations. Future research should focus on APD current established guidelines if we are to have more solid outcomes with larger samples as well an added focus on adults that are under-researched and possibly undermanaged.

## Data availability statement

The original contributions presented in the study are included in the article/supplementary material, further inquiries can be directed to the corresponding authors.

## Author contributions

MS, HT-V, MP, EG, EV, SM, PR, HG, NU, AS, D-EB, and VI contributed to conception and design of the study. MS, EG, D-EB, and VI organized the database. MS performed the statistical analysis. MS, D-EB, and VI wrote the first draft of the manuscript. EG wrote sections of the manuscript. All authors contributed to manuscript revision, read, and approved the submitted version.

## Conflict of interest

Author MS received honoraria as a consultant or for lectures for Lundbeck and Viatris.

The remaining authors declare that the research was conducted in the absence of any commercial or financial relationships that could be construed as a potential conflict of interest.

## Publisher’s note

All claims expressed in this article are solely those of the authors and do not necessarily represent those of their affiliated organizations, or those of the publisher, the editors and the reviewers. Any product that may be evaluated in this article, or claim that may be made by its manufacturer, is not guaranteed or endorsed by the publisher.
